# The Open Cryotop System Is Effective for the Simultaneous Vitrification of a Large Number of Porcine Embryos at Different Developmental Stages

**DOI:** 10.3389/fvets.2022.936753

**Published:** 2022-06-22

**Authors:** Alejandro Gonzalez-Plaza, Josep M. Cambra, Inmaculada Parrilla, Maria A. Gil, Emilio A. Martinez, Cristina A. Martinez, Cristina Cuello

**Affiliations:** ^1^Department of Medicine and Animal Surgery, Faculty of Veterinary Medicine, International Excellence Campus for Higher Education and Research (CMN), University of Murcia, Murcia, Spain; ^2^Institute for Biomedical Research of Murcia (IMIB-Arrixaca), Murcia, Spain; ^3^Department of Biomedical and Clinical Sciences (BKV), Division of Children's and Women's Health/Obstetrics and Gynaecology, Faculty of Medicine and Health Sciences, Linköping University, Linköping, Sweden

**Keywords:** vitrification, blastocyst, morula, Cryotop, SOPS, pig, embryo cryopreservation

## Abstract

The Superfine Open Pulled Straw (SOPS) system is the most commonly used method for vitrification of pig embryos. However, this system only allows the vitrification of four to seven embryos per straw. In this study, we investigated the effectiveness of the open (OC) and closed (CC) Cryotop^®^ systems to simultaneously vitrify a larger number of porcine embryos. Morulae, early blastocysts and full blastocysts were vitrified with the open Cryotop^®^ (*n* = 250; 20 embryos per device) system, the closed Cryotop^®^ (*n* = 158; 20 embryos per device) system and the traditional superfine open pulled straw (SOPS; *n* = 241; 4–7 embryos per straw) method. Fresh embryos from each developmental stage constituted the control group (*n* = 132). Data expressed as percentages were compared with the Fisher's exact test. The Kruskal-Wallis test was used to analyze the effect of the different vitrification systems on the embryo quality parameters and two-by-two comparisons were accomplished with the Mann-Whitney U test. Differences were considered statistically significant when *p* < 0.05. Vitrified and control embryos were incubated for 24 h and examined for viability and quality. At the warming step, the embryo recovery rate for the CC system was 51%, while all embryos were recovered when using OC and SOPS. There were no differences between the vitrification and control groups in the postwarming viability of full blastocysts. In contrast, morulae and early blastocysts that were vitrified-warmed with the SOPS system had lower viability (*p* < 0.01) compared to those from the OC, CC and control groups. The embryonic viability was similar between the OC and control groups, regardless of the developmental stage considered. Moreover, the embryos from the OC group had comparable total cell number and cells from the inner cell mass and apoptotic index than the controls. In conclusion, the OC system is suitable for the simultaneous vitrification of 20 porcine embryos at different developmental stages and provides comparable viability and quality results to fresh embryos subjected to 24 h of *in vitro* culture.

## Introduction

Unlike cattle, *in vivo*-derived embryos at the morula and blastocyst stages are the preferred source of embryos for commercial embryo transfer (ET) in pigs (1). High farrowing rates have been reported after transfer of these embryonic developmental stages (2–9) and, therefore, they are used for high-quality genetic embryo cryopreservation programs.

Cryopreservation by slow freezing has been shown to be ineffective in pig embryos because of their high content of intracellular lipids. Although the amount and composition may vary slightly between different breeds of *Sus scrofa domesticus*, vitrification is currently the main method for cryopreservation of pig embryos (10, 11). The ultrarapid vitrification systems are the most effective systems for vitrification of porcine embryos (12–14). These systems, which use a minimal volume of vitrification solution, achieve cooling rates as high as 20,000 °C/min (15). Among them, open pulled straw (OPS) (14) has been widely used for the cryopreservation of porcine embryos, specifically, the superfine open pulled straw (SOPS) (16). The SOPS vitrification of morulae and blastocysts has resulted in elevated embryo survival rates (17–20) and satisfactory fertility (~70%) after ET (7, 17). However, the SOPS system only allows the vitrification of small groups of embryos per straw (four to seven embryos) while maintaining the minimal volume of vitrification solution (1–2 μl) required for an adequate cooling rate (6, 17, 19, 21, 22). This is a disadvantage of this system for efficient cryopreservation and transfer of porcine embryo. A potentially successful ET requires between 30 and 40 vitrified *in vivo*-derived embryos, depending on whether the transfer is performed by surgical or nonsurgical procedures (7). This fact means that six to eight SOPS straws need to be warmed to accomplish a single ET. This technical limitation of the SOPS procedure can be overcome by using other vitrification systems, such as Cryotop^®^ [Kitazato, BioPharma Corporation, Ltd, Japan; (12)], which, thanks to its design, permits the simultaneous vitrification and warming of a greater number of embryos. Despite this technical advantage for polytocous species, very little information is available on the number of embryos that can be successfully vitrified at one time using the Cryotop^®^ system. In porcine species, although some studies have reported the vitrification of five to six *in vivo*-derived blastocysts loaded onto the tip of a Cryotop^®^ device (23–25), to the best of our knowledge, the simultaneous vitrification of a greater number of morulae and blastocysts using this system has not been investigated.

On the other hand, open vitrification systems (such as SOPS and Cryotop^®^) permit direct contact between the vitrification solution and liquid nitrogen (LN_2_), which may pose a potential risk of pathogen transmission during long-term storage (26–28). A solution to this concern would be to use sterile LN_2_, but this possibility is expensive or requires special equipment. The use of closed vitrification systems is an ideal alternative to prevent direct contact between the medium and LN_2_, thus preventing possible contamination of the embryos. Several closed vitrification systems, such as closed 0.25 ml straws (29) and CryoTip™ and CryoBio™'s high security vitrification systems (30), have been used to vitrify *in vitro*-produced porcine embryos, but the efficiency of closed vitrification of *in vivo*-derived embryos remains to be elucidated. Moreover, although several studies using *in vivo*-derived mouse (31, 32) and bovine (33) embryos have compared the efficiency of open and closed vitrification systems, no comparative information for *in vivo*-derived porcine embryos is available.

The aim of this study was to determine the effectiveness of open Cryotop^®^ (OC) and closed Cryotop^®^ (CC) systems for simultaneous vitrification in a single storage device of 20 *in vivo*-derived porcine morulae or blastocysts and to compare the postwarming embryo survival and quality obtained with these systems with the conventional SOPS procedure and also to non-vitrified control group.

## Materials and Methods

### Animals

The animals used as embryo donors for this experiment were sows (Landrace x Large White; 2 to 7 parity). The sows were housed in individual crates in an automatically ventilated room located in a commercial farm in southeastern Spain (Agropor S.L., Murcia, Spain). They were fed a commercial ration twice daily, and water was provided *ad libitum*.

### Superovulation, Estrous Detection and Artificial Insemination

Synchronization of the sows was performed by weaning. Superovulation was induced with 1,000 IU eCG (Folligon^®^, Intervet International B.V., Boxxmeer, the Netherlands; im) 24 h postweaning and 750 IU (hCG; Veterin Corion^®^, Divasa Farmavic, S.A., Barcelona, Spain; im) 2–3 days later.

Estrous detection was performed once daily in the morning (7:00 am) by using a mature vasectomized boar. Sows with a standing estrous reflex were inseminated immediately after estrous was detected and again 24 h later by intracervical artificial insemination with 3 × 10^9^ spermatozoa in 90 ml doses prepared at a commercial artificial insemination center using semen from an adult Duroc boar extended in Beltsville Thawing Solution extender (34).

### Embryo Collection

Embryos were collected on Day 6 (Day 0: onset of estrous) as previously described (3). Briefly, the donors were sedated and anesthetized with azaperone (2 mg/kg, im) and sodium thiopental (7 mg/kg, iv), respectively, and maintained with isoflurane (3.5–5%). After exposition of the ovaries and uterine horns, the number of corpora lutea on each ovary was counted and the embryos were recovered from each uterine horn with 30 ml of Tyrode's lactate-polyvinyl alcohol buffered with HEPES (TL-HEPES-PVA) (3, 35). Only embryos at the morula and blastocyst stages with good or excellent morphology (36), were selected for the experiment. The collected embryos were placed in 1 ml of TL-HEPES-PVA and transported at 39 °C to the laboratory at the University of Murcia.

### Vitrification and Warming

Vitrification of the embryos was performed within 3 h after collection as previously reported (8, 17). For this purpose, three vitrification systems were used: OC, CC, and SOPS. Embryos were vitrified into groups of 20 (OC and CC systems) or four to seven (SOPS system) embryos. The base medium for vitrification and warming was TL-HEPES-PVA. Embryo handling was performed at room temperature (RT; 22°C to 24°C), and the media were maintained at 38–39°C.

The embryos were washed twice in TL-HEPES-PVA and equilibrated in the first vitrification medium (V1: TL-HEPES-PVA + 7.5% ethylene glycol + 7.5% DMSO) for 3 min and then in the second vitrification medium (V2: TL-HEPES-PVA + 16% ethylene glycol + 16% DMSO + 0.4 M sucrose) for 1 min.

For SOPS vitrification, the embryos were transferred to a 1–2 μl drop of V2 medium in the final step and loaded into the straw by capillary action. Then, the straws were immersed horizontally into LN_2_. In the OC and CC groups, the embryos were transferred to a 40 μl droplet of V2 medium and then placed in groups of 1–3 embryos in 0.5–1 μl of V2 medium and loaded with a pulled glass pipette on the top of the polypropylene sheet of the Cryotop^®^ device. The total number of embryos vitrified in each device was 20. For the OC vitrification system, the device was immersed in LN_2_ and then covered with a plastic sheath before storage. For the CC vitrification system, the polypropylene sheet with the embryos was inserted into an LN_2_ precooled plastic sheath, and then the plastic sheath was heat sealed to avoid any direct contact between the sample and LN2. Vitrified embryos were kept in LN_2_ containers for 1 week before warming.

The embryos were warmed using the one-step dilution method (37). For the SOPS system, the end of the straw containing the embryos was immersed in 800 μl of warming medium (TL-HEPES-PVA with 0.13 M sucrose). In the OC and CC groups, the polypropylene sheet containing the embryos was immersed 2 ml of warming medium. Once the embryos were recovered, they were equilibrated in warming medium for 5 min and then washed in TL-HEPES-PVA. After that, the embryos were cultured for 24 h in 500 μl of NCSU23 medium supplemented with 0.4% BSA and 10% FCS at 39°C in an atmosphere of 5% CO_2_ in air while covered with paraffin oil (38).

### Recovery Rate at Warming and Assessment of Embryo Survival Rate After Culture

The recovery rate at warming was calculated as the number of embryos recovered after warming to the total number of vitrified embryos. The postwarming *in vitro* embryo viability was morphologically assessed at the end of culture using a stereomicroscope. Postwarmed morulae that developed to blastocysts and postwarmed blastocysts that restructured their blastocoelic cavities and had good or excellent morphology were considered viable. Control embryos (not vitrified) that continued their development during *in vitro* culture and exhibited good or excellent morphology were also considered viable. The survival rate was the ratio between the total number of viable embryos at the end of the culture and the total number of embryos cultured.

### Differential Staining of the Embryos

**Figure 1:**
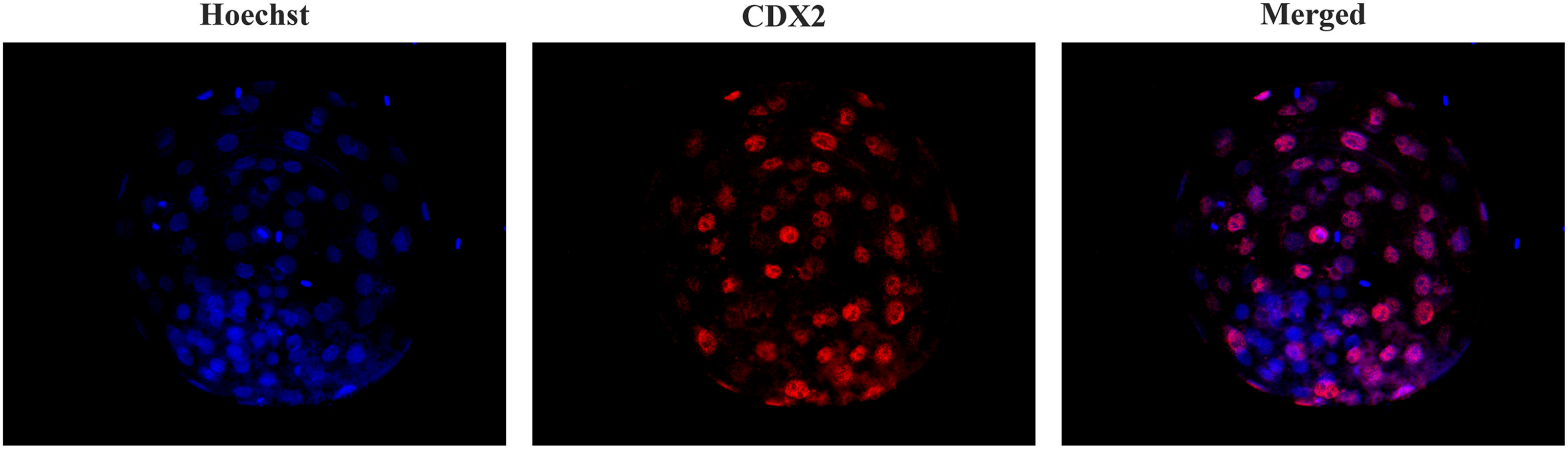
Differential staining of blastocysts. Embryos were stained with Hoechst-33342 (blue fluorescence) for total cell staining and anti-CDX2 (red fluorescence) for trophectoderm cell staining. The merged image shows blue and pink fluorescence for inner cell mass nuclei and TE nuclei, respectively.

To assess the total cell number (TCN), inner cell mass (ICM) and trophectoderm (TE) cells, some viable blastocysts were differentially stained after *in vitro* culture as previously described (39). For this purpose, the embryos were fixed by immersion in 4% paraformaldehyde in PBS for 30 min at RT. After fixation, the embryos were transferred to a 500 μl drop of PBS containing 0.3% BSA (PBS-BSA). Unless otherwise indicated, all washes were performed three times with PBS-BSA for 2 min. The embryos were permeabilized overnight in PBS containing 1.5% Triton X-100 and 0.15% Tween 20 (PBS-TT). After permeabilization, they were washed in PBS-BSA and denatured by sequential exposure for 20 min to HCl medium (2 N) and for 10 min to Tris HCl medium (100 mM). After denaturation and washing, the embryos were equilibrated in blocking solution (PBS containing 1% BSA, 10% normal donkey serum and 0.05% Tween 20) for 6 h. Then, the embryos were washed and incubated for 36 h in the dark with 1 μg/ml anti-CDX2 primary antibody (BioGenex, Molenstraat, The Hague, The Netherlands) in PBS-BSA at 4-6 °C. After incubation with the primary antibody, the embryos were washed and incubated for 1 h with 2 μg/ml donkey anti-mouse IgG Alexa Fluor^®^ 568 conjugate (ThermoFisher Scientific, Eugene, Oregon, USA) in blocking solution and then washed again. Finally, the embryos were placed on a slide in Vectashield (Vector, Burlingame, USA) containing 10 μg/ml Hoechst-33342 and covered and flattened with a coverslip. Stained blastocysts were evaluated with a fluorescence microscope (excitation filter of 400–440 nm) to visualize TCN, whose nuclei displayed blue fluorescence, and an excitation filter of 510–560 nm to visualize TE cells, whose nuclei showed red fluorescence (Figure 1). The number of ICM cells was calculated as the difference between the number of TE and TCN cells. The ICM/TCN ratio was considered as the ratio between the number of nuclei in the ICM to the total number of nuclei.

### Cellular Apoptosis

**Figure 2:**
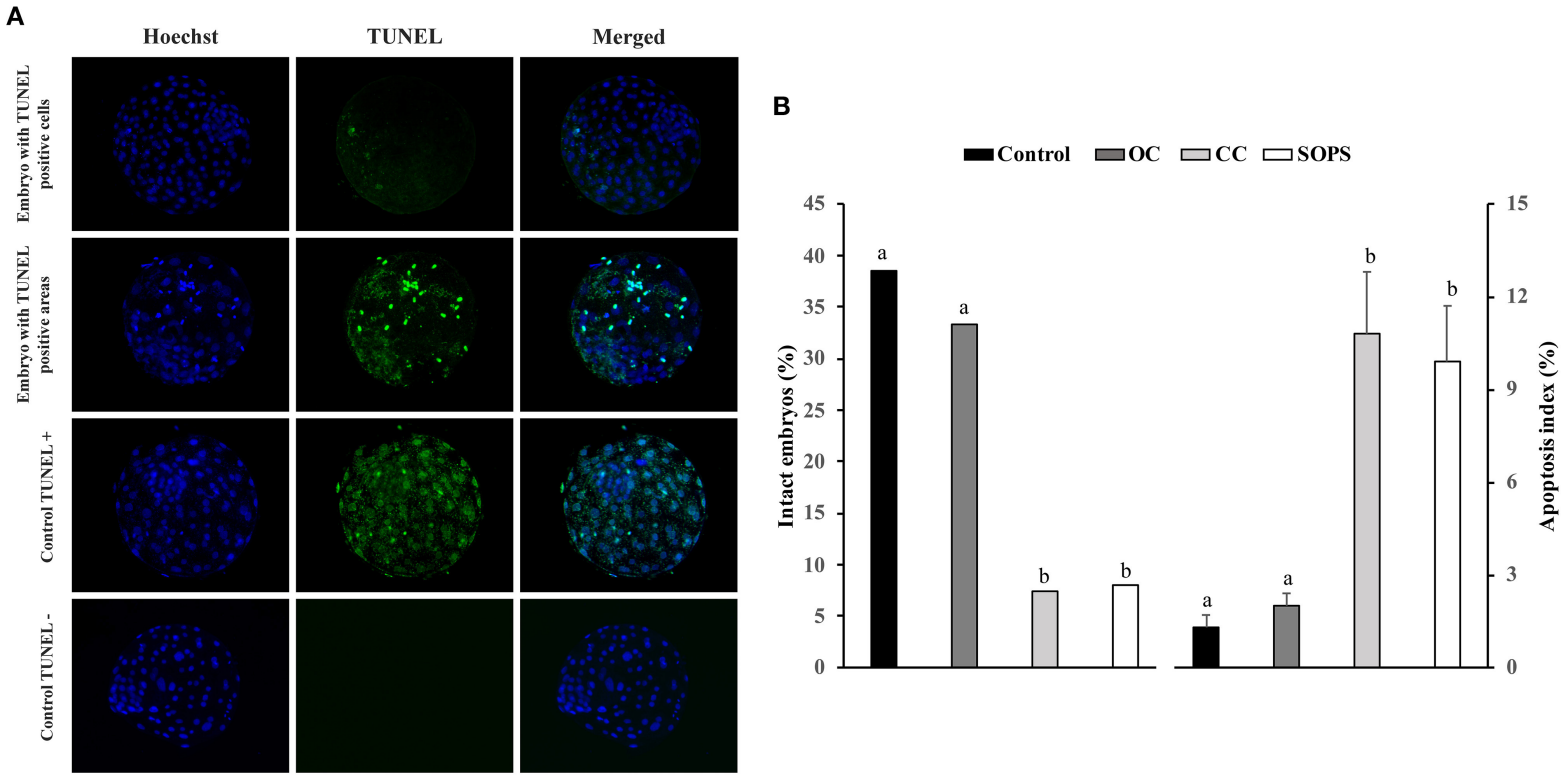
Apoptosis in vitrified blastocysts. **(A)** Representative fluorescence images of the TUNEL assay of blastocysts stained with Hoechst-33342 (blue fluorescence) for total cells and TUNEL labeling (green fluorescence) for the detection of apoptosis. Positive and negative controls are included. **(B)** Apoptosis levels in blastocysts from control (*n* = 26), open Cryotop^®^ (OC; *n* = 24), closed Cryotop^®^ (CC; *n* = 27) and SOPS (*n* = 25) groups. a,b, *p* < 0.001.

The evaluation of apoptosis in the blastocysts was performed with a cell death detection method based on the terminal deoxynucleotidyl transferase (TdT)-mediated dUTP nick-end labeling (TUNEL) technique as previously reported (40, 41). This technique was performed using the Apoptosis BrdU TUNEL Assay Kit (A23210; Thermo Fisher Scientific). The embryos were first fixed as described above. Unless otherwise indicated, all washes in this technique were performed in triplicate with PBS-BSA for 5 min. After fixing and washing, embryos were permeabilized overnight in PBS-TT and washed in PBS-BSA for 10 min. Some fixed and permeabilized embryos were used as positive and negative controls. After permeabilization, positive control blastocysts were incubated with DNase I (10 μg/ml) in PBS-BSA for 20 min at 39°C in the dark. Subsequently, all blastocysts were washed in 0.5% Tween 20 in PBS-BSA for 10 min, incubated with TdT enzyme and Br-dUTP (5-bromo-2'-deoxyuridine 5'-triphosphate), and then covered with paraffin oil in 10 μl drops for 1 h at 39°C. Negative control blastocysts were incubated in the same medium and under the same conditions described but without TdT enzyme. After incubation, the embryos were washed and transferred to rinse buffer containing a mouse anti-BrdU-Alexa Fluor^®^ 488 conjugate for 30 min at RT. The blastocysts were then washed and placed on a slide in Vectashield (Vector, Burlingame, USA) containing 10 μg/ml Hoechst-33342 and covered and flattened with a coverslip. Samples were examined with a fluorescence microscope (excitation filter of 400–440 nm) to visualize the TCN, whose nuclei displayed blue fluorescence, and a 465–495 nm excitation filter to visualize TUNEL-positive nuclei, which exhibited green fluorescence (Figure 2). The apoptotic index was defined as the ratio between the number of apoptotic cells and the total number of cells in each embryo. Those embryos without TUNEL-positive nuclei were considered intact. Embryos that displayed TUNEL-positive areas were distributed into two groups: embryos with TUNEL-positive areas that occupied < 20% of the embryo surface and embryos showing TUNEL-positive areas that occupied between 20 and 50% of the embryo surface.

### Experimental Design

Embryos were retrieved from 40 sows and pooled into groups depending on their developmental stage: morulae (*n* = 299), early blastocysts (*n* = 325) and full blastocysts (*n* = 157). A portion of embryos from each stage of development were cultured for 24 h to assess the *in vitro* development of the fresh embryos (control; *n* = 132). The rest of the embryos from each developmental stage were vitrified using different vitrification systems: OC (*n* = 250), CC (*n* = 158), and SOPS (*n* = 241). The recovery rate of each vitrification system was calculated immediately after warming, and the recovered embryos were cultured for 24 h to assess the embryo viability. After viability assessment, some blastocysts from the control and vitrification groups were processed for differential staining (*n* = 60) or cellular apoptosis assessment (*n* = 89).

### Statistical Analysis

Data were analyzed using IBM SPSS software, version 24.0 (SPSS; Chicago, IL, USA). All data expressed as percentages were compared using the Fisher's exact test. The normality of the variables was tested by the Kolmogorov–Smirnov test. The nonparametric Kruskal-Wallis test was used to analyze the effect of the different vitrification systems on the TCN, ICM, ICM/TCN ratio, mean number of apoptotic cells, and apoptotic index. When the test showed a significant effect, two-by-two comparisons were accomplished with the Mann-Whitney U test. Differences were considered statistically significant when *p* < 0.05. The results are expressed as percentages and mean values ± SEM.

## Results

### Embryo Collection

The average ovulation rate of the sows was 22.8 ± 3.3 corpora lutea (range from 15 to 30), with 829 structures recovered after surgical embryo collection (91.1% recovery rate), among which 94.1% were embryos and the rest were unfertilized oocytes and degenerated embryos. Among the 781 embryos recovered, 299 (38.3%) were morulae, 325 (41.6%) were early blastocysts and 157 (20.1%) were full blastocysts.

### Recovery Rate and Embryo Survival After Vitrification and Warming

While 77 out of 158 embryos (48.7%) were lost during vitrification and warming in the CC group, all vitrified embryos were recovered after warming in the OC and SOPS groups (*p* < 0.0001).

**Figure 3:**
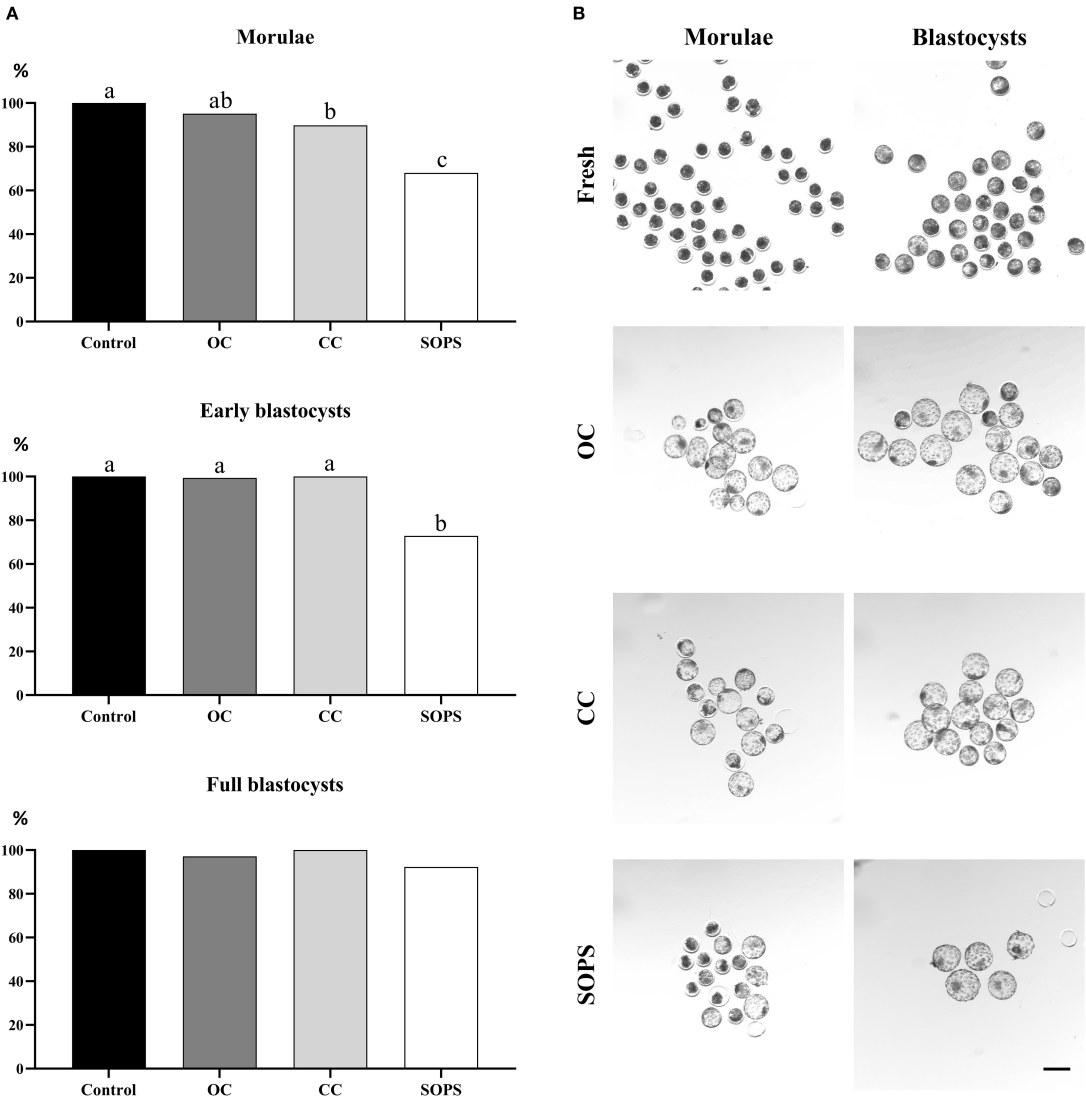
Embryo survival after vitrification and warming. **(A)** Postwarming survival rates of morulae (*n* = 283), early blastocysts (*n* = 283), and full blastocysts (*n* = 137) vitrified with open Cryotop^®^ (OC), closed Cryotop^®^ (CC) and SOPS systems. a,b,c *p* < 0.01. **(B)** Representative images of *in vivo*-derived morulae and blastocysts prior and after vitrification with the different systems. Scale bar: 200 μm.

The survival rates of the control and vitrified embryos at different developmental stages after 24 h of *in vitro* culture are shown in Figure 3. Survival rates were similar in the OC and CC groups, regardless of the embryonic stage, although survival rate for the CC group was lower (*p* < 0.01) than the control group for the morula stage. The SOPS group showed the lowest (*p* < 0.01) survival rate of all groups when only morulae or early blastocysts were considered. Full blastocysts from all vitrification groups showed high viability values, which were similar to those obtained in the control group.

### Total Cell Number, Differential Cell Count and Cellular Apoptosis

The embryos from the CC group showed lower (*p* < 0.05) TCNs and ICM values than their control counterparts. However, there were no differences in these parameters between the different vitrification groups. The number of TE cells and the ICM/TCN ratio were similar between the vitrified and control groups (Table 1).

**Table 1:** Total cell number and differential cell counts of blastocysts derived from embryos vitrified with open Cryotop^®^ (OC), closed Cryotop^®^ (CC) or SOPS systems.

**Groups**	***n***	**TCN**	**ICM**	**TE**	**Ratio**
Control	16	90.6 ± 4.7^a^	22.3 ± 2.4^a^	68.4 ± 4.5	24.9 ± 2.9
OC	13	72.2 ± 6.2^ab^	15.5 ± 1.8^ab^	56.7 ± 5.5	21.7 ± 2.3
CC	13	68.5 ± 5.9^b^	14.8 ± 2.0^b^	54.1 ± 4.5	21.0 ± 1.7
SOPS	18	80.8 ± 5.8^ab^	18.6 ± 1.7^ab^	62.2 ± 4.6	23.0 ± 1.4

*TCN, total cell number; ICM, inner cell mass; TE, trophectoderm*.

*Ratio: percentage of ICM cells to the TCN*.

*^a, b^Different superscripts indicate significant differences (p < 0.05). Data are expressed as the mean ± SEM*.

The percentage of intact embryos (with no apoptotic cells) was 3- to 5-fold higher (*p* < 0.05) and the apoptosis index was 5- to 8-fold lower (*p* < 0.05) in the OC and control groups than in the CC and SOPS groups (Figure 2). Additionally, the mean number of apoptotic cells per embryo from the CC and SOPS groups was higher (p < 0.001) than those from the OC and control groups (Table 2). Some embryos did not show well-defined TUNEL-positive cells but rather TUNEL-positive areas. The CC group had the highest (*p* < 0.05) number of embryos with TUNEL-positive areas compared with the OC and control groups. Additionally, embryos with TUNEL-positive areas between 20 and 50% of the embryo surface were only found in the CC and SOPS groups (Table 2).

**Table 2:** Results of the TUNEL assay of morulae and early blastocysts vitrified with open Cryotop^®^ (OC), closed Cryotop^®^ (CC) or SOPS systems.

**Groups**	***n***	**Mean number of apoptotic cells [Range of cells]**	**A1, *n* (%)**	**A2, *n* (%)**	**TA, *n* (%)**
Control	26	1.0 ± 0.3 [1–4]^a^	0 (0.0)	0 (0.0)	0 (0.0)^c^
OC	24	1.8 ± 0.6 [1–7]^a^	2 (8.3)	0 (0.0)	2 (8.3)^c^
CC	27	6.7 ± 1.2 [1–25]^b^	7 (25.9)	2 (7.4)	9 (33.3)^d^
SOPS	25	7.1 ± 1.0 [2–36]^b^	4 (16.0)	1 (4.0)	5 (24.0)^cd^

*A1: Embryos showing TUNEL-positive areas that occupied <20% of the embryo surface*.

*A2: Embryos showing TUNEL-positive areas that occupied between 20 and 50% of the embryo surface*.

*TA: Total number of embryos showing TUNEL-positive areas*.

*Different superscripts within a variable indicate significant differences (a,b, p < 0.001; c,d, p < 0.05)*.

## Discussion

The results of this study show that the OC is a suitable system for the simultaneous vitrification of 20 porcine embryos at the morula or blastocyst stages, which is of great importance for the practical application of embryo vitrification and transfer in swine. The possibility of simultaneously vitrifying a large number of embryos not only greatly simplifies the current vitrification protocols, but also facilitates the embryo warming and embryo transfer processes, which are normally performed under field conditions.

Although the Cryotop^®^ system is typically used for human oocytes and embryos (42, 43), where only 1–2 oocytes or embryos are vitrified at a time, this system is an ideal alternative for the vitrification of a large number of embryos in polytocous species, such as the pig. In our study, a total of 20 porcine embryos were successfully placed in the polypropylene sheet of the Cryotop^®^ device and vitrified, showing similar postwarming viability and quality as nonvitrified control embryos. The success of this vitrification system could be related to the minimal volume of medium surrounding the sample (44, 45). Based on mathematical models, recent results indicate that the number of embryos placed on an OC device does not have a great impact on cooling rates as long as the volume of the vitrification medium remains small (46). Our study supports this computational simulation. To vitrify 20 embryos, it is necessary to cover a large area of the Cryotop^®^ polypropylene sheet, but the embryos are placed in 0.5–1 μl droplets containing one to three embryos each, and therefore, each embryo is surrounded by a minimal volume of vitrification medium.

The postwarming recovery rate was significantly reduced in the CC group, where almost half of the embryos were lost during the vitrification and/or warming processes. This could be explained by the fact that the CC system is a closed system with a polypropylene film with an L-shaped tip to protect the sample when inserted into the external straw; since in our experiment we placed multiple droplets in the film, the protective effect might be inefficient, allowing the loss of embryos during the insertion of the device into the external straw before plunging into LN_2_. The high number of embryos lost with this system is a compelling reason to reject this method for the simultaneous vitrification of a large number of embryos.

The postwarming embryo viability results obtained in this study using the SOPS system are comparable to those previously reported for porcine morulae (19, 47) and blastocysts (17, 20, 48). It is generally assumed that morulae have a lower vitrification capacity than blastocysts, as most studies have found a lower survival rate for SOPS-vitrified morulae (19, 47) than for SOPS-vitrified blastocysts (17, 20, 48). The higher lipid content in the embryos at the morula stage compared to that in blastocysts has been related to their low cryotolerance (49). In this study, the use of the Cryotop^®^ systems improved the results obtained with morulae and early blastocysts compared with those of the SOPS method. Moreover, embryos vitrified with the OC system showed similar viability to their control counterparts.

In agreement with previous reports on mouse and bovine embryo vitrification (31, 33), our study showed similar survival rates between the CC and OC groups. However, CC embryos, such as SOPS embryos, presented a higher apoptosis index, more TUNEL-positive cells, and a lower percentage of intact embryos than OC and control embryos. It is well known that an increased apoptosis level in embryos is one of the main consequences of vitrification and that it is associated with lower embryo viability (41, 50). Our results are consistent with previous studies (51, 52), which reported that SOPS vitrification and warming increased apoptosis in porcine embryos. Interestingly, in the present study, the apoptosis parameters were low in embryos from the OC and control groups, with no differences between the groups. These results agree with previous studies using vitrified *in vitro*-produced embryos (53) and suggest that the apoptotic values observed in OC and control embryos can be considered physiological, since apoptosis is a natural phenomenon occurring in mammalian blastocysts (54).

The main advantage of the OC system compared to the CC and SOPS systems is that the contact of the sample with LN_2_ during vitrification and with the warming solution during warming is more direct, which should lead to higher cooling and warming rates, as has also been reported for other devices (44, 55). Achieving high cooling rates during vitrification is a key factor for success, as it helps to minimize chilling injury by reducing the exposure of embryos to critical temperatures (56, 57). In addition, several studies using mouse oocytes (58, 59) and embryos at different developmental stages (60, 61) suggest that the warming process may have a larger impact on embryo survival rates than the cooling step during vitrification. Therefore, the apparent increased cooling and warming rates using the OC system could explain the better outcomes in terms of embryo survival and/or apoptosis compared with the other two vitrification systems evaluated.

## Conclusion

In conclusion, the use of the OC is suitable for the simultaneous vitrification of at least 20 porcine embryos at the morula or blastocyst stage and yields similar postwarming results in terms of embryo survival, TCN, ICM, ICM/TCM ratio and apoptosis levels to those achieved in control embryos.

## Data Availability Statement

The original contributions presented in the study are included in the article/supplementary material, further inquiries can be directed to the corresponding authors.

## Ethics Statement

The study was reviewed and approved by the Ethics Committee for Experiments with Animals of the University of Murcia (Code: 486/2018).

## Author Contributions

CC, CM, and EM contributed to conception, design of the study, and reviewed and edited the manuscript. CC and EM directed the experiments. AG-P, JC, IP, MG, EM, and CC performed the experiments. AG-P, CC, CM, and EM performed the statistical analysis and analyzed and interpreted the data. AG-P and CC wrote the first draft of the manuscript. All authors contributed to manuscript read and approved the submitted version.

## Funding

This research was funded by the MCIN/AEI/10.13039/501100011033 and by ERDF A way of making Europe (RTI2018-093525-B-I00), Madrid, Spain, Fundacion Seneca (19892/GERM/15), Murcia, Spain, and the Swedish Research Council FORMAS (Projects 2019-00288), Stockholm, Sweden.

## Conflict of Interest

The authors declare that the research was conducted in the absence of any commercial or financial relationships that could be construed as a potential conflict of interest.

## Publisher's Note

All claims expressed in this article are solely those of the authors and do not necessarily represent those of their affiliated organizations, or those of the publisher, the editors and the reviewers. Any product that may be evaluated in this article, or claim that may be made by its manufacturer, is not guaranteed or endorsed by the publisher.
